# Establishment of primary mixed cell cultures from spontaneous canine mammary tumors: Characterization of classic and new cancer-associated molecules

**DOI:** 10.1371/journal.pone.0184228

**Published:** 2017-09-25

**Authors:** Luciana B. Gentile, Marcia K. Nagamine, Luiz R. Biondi, Daniel S. Sanches, Fábio Toyota, Tatiane M. Giovani, Isis P. de Jesus, Ivone I. M. da Fonseca, Nicolle Queiroz-Hazarbassanov, Bruno L. Diaz, Cristina de O. Massoco Salles Gomes, Maria Lucia Z. Dagli

**Affiliations:** 1 Laboratory of Experimental and Comparative Oncology, Department of Pathology, School of Veterinary Medicine and Animal Sciences, University of São Paulo, São Paulo, São Paulo, Brazil; 2 Veterinary Hospital Cães e Gatos, Osasco, São Paulo, Brazil; 3 Department of Pathology, School of Veterinary Medicine and Animal Sciences, University of São Paulo (USP), São Paulo, São Paulo, Brazil; 4 Applied Pharmacology and Toxicology Laboratory, School of Veterinary Medicine and Animal Sciences, University of São Paulo, São Paulo, São Paulo, Brazil; 5 Laboratory of Inflammation, Carlos Chagas Filho Biophysics Institute (IBCCF), Federal University of Rio de Janeiro (UFRJ), Rio de Janeiro, Rio de Janeiro, Brazil; University of Wisconsin Madison, UNITED STATES

## Abstract

There are many factors which make canine cancer like cancer in humans. The occurrence of spontaneous mammary tumors in pet dogs, tumor genetics, molecular targets and exposure to the same environmental risk factors are among these factors. Therefore, the study of canine cancer can provide useful information to the oncology field. This study aimed to establish and characterize a panel of primary mixed cell cultures obtained from spontaneous canine mammary tumors. Eight established cell cultures obtained from one normal mammary gland, one complex adenoma, one mixed adenoma, two complex carcinomas and two mixed carcinomas were analyzed. The gene expression levels of classic molecular cancer players such as fibroblast growth factor receptor (*FGFR*) *2*, breast cancer (*BRCA*) *1*, *BRCA2* and estrogen receptor (*ESR*) *1* were evaluated. For the first time, three orphan nuclear receptors, estrogen-related receptors (*ERRs*) *α*, *β* and *γ* were studied in canine mammary cancer. The highest expression level of *ERRα* was observed in complex carcinoma-derived cell culture, while the highest levels of *ERRβ* and *γ* were observed in cells derived from a mixed carcinoma. Meanwhile, complex carcinomas presented the highest levels of expression of *ESR1*, *BRCA1* and *FGFR2* among all samples. BRCA2 was found exclusively in complex adenoma. The transcription factor *GATA3* had its highest levels in mixed carcinoma samples and its lowest levels in complex adenoma. Proliferation assays were also performed to evaluate the mixed cell cultures response to ER ligands, genistein and DES, both in normoxia and hypoxic conditions. Our results demonstrate that morphological and functional studies of primary mixed cell cultures derived from spontaneous canine mammary tumors are possible and provide valuable tool for the study of various stages of mammary cancer development.

## Introduction

The availability of pre-clinical animal models for human breast cancer represents a relevant challenge in the research of this type of neoplasm. *In vivo* models with transgenic mice or tumor-bearing mice with human neoplastic cell lines, although widely used, have failed in reproducing some aspects of human breast cancer such as heterogeneity, tumor microenvironment and steroid hormone-dependent growth [1, 2]. Beyond the evolutionary distance between humans and mice, additional differences can be originated from induced genetic modifications, as in transgenic mice, or from the modified presence of components of normal adjacent tissue, as in the case of tumor transplants in mice [3, 4, 5, 6]. Therefore, the limitation of mice models for the oncology field is an obstacle to overcome.

One alternative for this issue relies on the fact that naturally occurring cancers in pet dogs and humans share many features, including histological appearance, tumor genetics, molecular targets, biological behavior and response to conventional therapies [7, 8, 9, 10, 11]. Studying dogs with cancer is likely to provide a valuable perspective that is distinct from that generated by the study of rodent cancers alone. For many families of genes, particularly those associated with cancer, the similarities between gene sequences of dog and man are closer than the ones in mice [12]. In mammary carcinomas, the modified expression of genes *ERBB2* and *TP53* was similar in the two species, suggesting similar functions in carcinogenesis [13, 14, 15]. Moreover, comparative histologic analyses indicate that the observed intra-tumor heterogeneity in human mammary tumors also occurs in canine tumors [16].

The natural consequences of this heterogeneity are some aspects of unfavorable prognoses in cancer, such as acquired resistance to therapy, recurrence and metastasis. In contrast to mice generated in the laboratory, dogs share the environment with human beings and are exposed to some of the same carcinogens [17]. Furthermore, as in human beings, treatment with progesterone, advanced age, obesity and diet, also represent risk factors for the development of breast cancer in dogs [18].

Various studies have focused on new signaling pathways, mediated by estrogen-related receptors (ERRs) in the development of breast cancer. ERRs are constitutively active receptors that share a high degree of homology with classical estrogen receptors (ERs). However, they do not bind to estrogen, while ERs do [19, 20]. ERRs are involved in the development of alternative pathways that lead to the development of cancer and are regarded as potential therapeutic targets for the treatment of breast cancer that do not respond to conventional therapies. The ERR family is composed of three members, ERRα (or NR3B1), ERRβ (or NR3B2) and ERRγ (or NR3C3) [19, 21, 22, 23, 24]. ERRs are orphan nuclear receptors with no natural ligands identified. However, the crystallographic structure of ERα with its natural ligand indicated that many amino acid residues crucial for the recognition of estradiol are conserved between the members of the families of ERs and ERRs [25], suggesting that some ligands of ER and ERR could be structurally related. Recently, the functions of ERRs have been studied in human mammary and endometrial carcinomas [19, 26, 27, 28, 29], ovarian cancer [30], and prostate cancer [31], including cell lines and tissues.

*BRCA1* and *BRCA2* genes code for proteins located in the cell nucleus and play an essential role in the structural maintenance and numerical stability of chromosomes during cellular division. Chromosomal instability initiated by the inactivation of *BRCA* genes is the main cause of carcinogenesis in the mammary gland due to its involvement with DNA repair and regulation of cytokinesis [32, 33]. Considered tumor suppressor genes, the inactivation of *BRCA1* and *BRCA2* after loss of heterozygosity confer a risk of 56 to 87% of development of breast cancer in women and somatic mutations of *BRCA2* are associated with aggressive metastasis in lymph nodes in humans [34].

GATA-3 is a transcription factor that orchestrates the gene expression profile of several tissues during embryogenesis. Particularly in the mammary tissue, this gene is responsible for the differentiation of luminal cells in mammary glands. Considered a possible tumor suppressor gene, *GATA-3* appears to control differentiation and proliferation of mammary cancer cells, since the majority of human breast tumors originate from epithelial luminal cells [35, 36]. Experimental studies with mice demonstrate that the loss of *GATA-3* expression is associated with high histologic grade tumors, positive lymph nodes, absence of estrogen and progesterone receptors with *HER2* overexpression. On the other hand, its presence is enough to induce the tumor differentiation and to avoid the dissemination of breast cancer, characterizing this marker as strong predictive factor and independent from the clinical outcome in this type of cancer [37].

The goal of the present study was to characterize the expression of cancer progression related molecules in primary mixed cell cultures obtained from spontaneous canine mammary tumors. Tumors were classified according to the criteria proposed previously [38, 39]. Briefly, tumors were divided into two histological categories, adenomas and carcinomas, with subtypes named simple, complex or mixed. Adenomas display well-demarcated noninfiltrative nodular lesions while carcinomas display epithelial malignant proliferation. The differences between the subtypes are the presence of benign proliferation of myoepithelial cells (complex tumors) and/or presence of cartilage and/or bone and/or adipose tissue, possibly in combination with fibrous tissue (mixed tumors). The isolation method used in this study allowed the separation of fibroblasts from the epithelial/myoepithelial component of mammary tissues, responsible for the origin of adenomas and carcinomas. Therefore, each primary cell culture represent a different histological type and, as consequence, its respective environment complexity, preserving the interactions between epithelial and myoepithelial cells.

## Materials and methods

### Samples

Tissue samples were obtained in Veterinary Hospital, School of Veterinary Medicine, Universidade Anhembi Morumbi, São Paulo and Hospital Veterinário Cães e Gatos, Osasco, São Paulo, Brazil. The breeds of female dogs used in this study were Poodle, Dachshund, Yorkshire Terrier, German Shepherd and free-ranging urban dog at ages of 7, 8, 10 and 13 years old. The mammary gland lesions were surgically removed for diagnosis and therapeutic purposes and tissue samples were provided for the current study with permission of the dog owners. All lesions were classified according to the criteria proposed previously [38, 39]. Seven primary mixed cell cultures were established from seven spontaneous canine mammary tumors in distinct animals: two complex carcinomas, two mixed carcinomas, one complex adenoma and one mixed adenoma. One mixed cell culture was established from normal mammary glands from one non-cancer host female dog and used as control in the experiments. The epithelial normal mammary cells were obtained from abdominal and inguinal mammary glands of a female dog, 10 years old, breed Labrador Retriever, whose cause of death was intestinal torsion (cancer non-related disease).

The School of Veterinary Medicine and Animal Sciences of the University of São Paulo, Brazil approved the protocols for this study (protocol number 2323/2011) according to the ethical principles in Animal Research adopted by the Ethics Committee in the use of animals.

### Isolation of primary mixed cell cultures from spontaneous mammary lesions in canines

The isolation method consisted of incubation at 37°C of the tissue sample in a solution of collagenase type IV (1 mg/ml) (Sigma) and hyaluronidase (1 mg/ml) (Sigma) followed by digestion with trypsin-EDTA (0.25%) (Gibco). All enzymes were diluted in HBSS containing antibiotics (200 U/ml penicillin, 200 μg/ml streptomycin, and 25 μg/ml amphotericin B). Tumor fragments were digested in 10 volumes of tumor digestion solution for about 2 h at 37°C with periodic agitation. After digestion, trypsin was then inactivated with media containing fetal bovine serum (FBS) and the sample centrifuged (1500 rpm for 10 min) to remove the enzyme solution, washed with HBSS, and centrifuged again (1500 rpm for 10 min). Cells were cultured in Advanced DMEM (Gibco) containing HEPES (10 mM), Glutamax (Gibco), antibiotics (200 U/ml penicillin, 200 μg/ml streptomycin, and 25 μg/ml amphotericin B), cholera toxin (10 ng/mL), MEGS (Mammary Epithelial Growth Supplement, Gibco) and 0.5% of inactivated fetal bovine serum. Cells were maintained in atmosphere of 5% CO_2_ at 37°C. This method of obtaining cells provided organoids, epithelial and stromal cells in the same cell culture.

Differential trypsinization technique was performed after the cells reached partial confluency [40]. This technique is based on the different adhesion capacities of stromal and epithelial cells. The culture medium was removed and the flask washed with PBS-EDTA for 2 min to remove traces of FBS. Then, differential trypsinization was initiated. Cells not adhered and organoids were not dismissed and returned to cultivation. This technique involves placing the cells in contact with trypsin-EDTA solution 0.25% (Gibco) for 10 to 15 min, collecting the supernatant (overall, the fraction that comes off before are stromal cells), growing it in another bottle and maintaining the remaining cells (epithelial cells in majority) cultured in the original bottle. The bottle was rinsed with FBS-containing medium to inactivate the trypsin. All primary cell cultures were submitted three times to differential trypsinization before being used in experiments and were kept until the ninth passage. The phenotype characterization of the mixed cell cultures was performed by qPCR using oligos for MUC1 (Cf02626760_m1, Cf02680908_s1) an epithelial marker, and MYH11 (Cf02697091_m1, Cf02632489_m1) a smooth muscle marker.

The differential trypsinization has been chosen to obtain mesenchymal cells-free primary cultures to isolate the epithelial counterpart of the tissue (epithelial and myoepithelial cells).

The primary mixed cell cultures were analyzed with an inverted microscope (Motic AE31 Elite, Motic) and image acquisition was performed with Motic Images Plus 2.0 software (Motic China Group, 2007).

### Analysis of DNA ploidy, cell cycle and proliferation by flow cytometry

For the analysis of DNA ploidy and cell cycle, cells were grown, harvested and counted. One million cells were fixed in 70% ethanol, followed by staining with propidium iodide (PI) diluted in PBS pH 7.4 (PI 200 μg/ml, RNAse A 20 mg/ml, 0.1% Triton X-100) for 15 min at 37°C. For proliferation assays harvested cells were counted and 1x10^6^ were stained with 5μM Carboxyfluorescein diacetate succinimidyl ester (CFDA-SE, Sigma-Aldrich), for 30min at 37°C with shaking. The reaction was stopped with two sequential wash steps with complete culture media and cells returned to culture afterwards. All samples were acquired in a FACSCalibur (Becton Dickinson, Burlington, MA) flow cytometer equipped with CellQuest Pro Software, (Becton Dickinson, San Jose, CA) using the FL1 (CFDA-SE) and FL2 (PI) channels. Analyses of DNA ploidy, cell cycle and proliferation were performed using the FlowJo software version 7.6.4 (Tree Star Inc, San Carlos, CA).

### RNA extraction

The primary neoplastic cells obtained from spontaneous mammary tumors were processed according to the instructions of manufacturer (TRIzol—Life Technologies). The purified RNA was then quantified and its integrity analyzed using NanoDrop spectrophotometer 2000 (ThermoScientific).

### Real time quantitative PCR (qPCR)

Total RNA isolated as above was further treated with an RNase-free DNase set (Qiagen) to eliminate possible contaminating genomic DNA. cDNA was synthesized from the total RNA preparation using the SuperScript II Reverse Transcriptase kit (Invitrogen) according to the instructions of the manufacturer. qPCR was performed by using TaqMan gene expression assays and Universal PCR Master Mix (Applied Biosystems, Foster City, CA), with the following protocol: denaturation by a hot start at 95°C for 10 min, followed by 40 cycles of a 2-step program (denaturation at 95°C for 15 s and annealing/extension at 60°C for 1 min). Assay numbers were as follows: FGFR2 (Cf02623903_m1, Cf02623899_m1), BRCA1 (Cf02625915_g1, Cf02625925_m1), MUC1 (Cf02626760_m1, Cf02680908_s1), BRCA2 (Cf02622077_m1, Cf02622063_m1), MYH11 (Cf02697091_m1, Cf02632489_m1), ESR1 (Cf02624844_m1, Cf02624844_m1), ESRRA (Cf02626582_gH), ESRRB (Cf02681395_s1, Cf02681401_s1), ESRRG (Cf00976241_m1, Cf00976243_m1) and B2M (Cf02659079_m1). RNA samples were treated with DNAse (Deoxyrinonuclease I, Invitrogen) and reverse transcribed using premix SuperScript VILO™ Master Mix (11755050 Life Technologies) to obtain cDNA. The data were analyzed by the comparative delta Ct method. The values of Ct obtained in these experiments were used to calculate the relative expression of mRNA of each gene of interest relative to a housekeeping gene, β-2-microglobulin (B2M –code number Cf02659079_m1), for normalization. This value is called the Delta Ct (ΔCt) and is calculated by the formula: ΔCt = (Ct target gene − Ct endogenous control). Due to the logarithmic characteristic of this variable, the parameter 2^-ΔCt^ was used for the analysis of data [41].

### Statistical analysis

The statistical analysis of the differences was performed using two-way ANOVA with GraphPadPrism version 5. Parallel experiments are used to determine significance and values of *P* < 0.05 are considered statistically significant.

## Results

**Fig 1:**
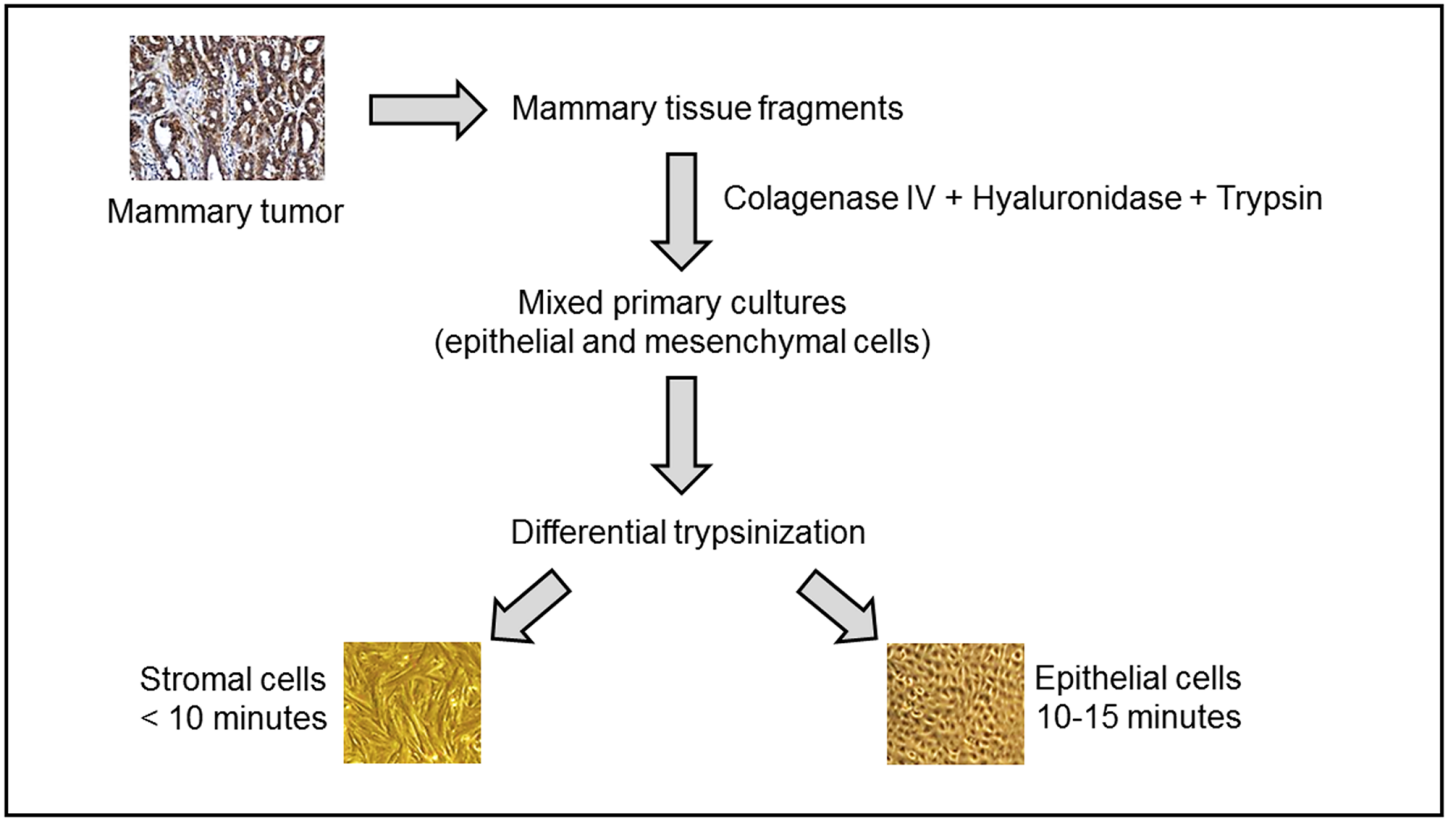
Scheme of culture method. Schematic illustration of the method of obtaining mixed culture from the gross tumor containing all cell types present in the breast tissue and further purification with differential trypsinization.

**Fig 2:**
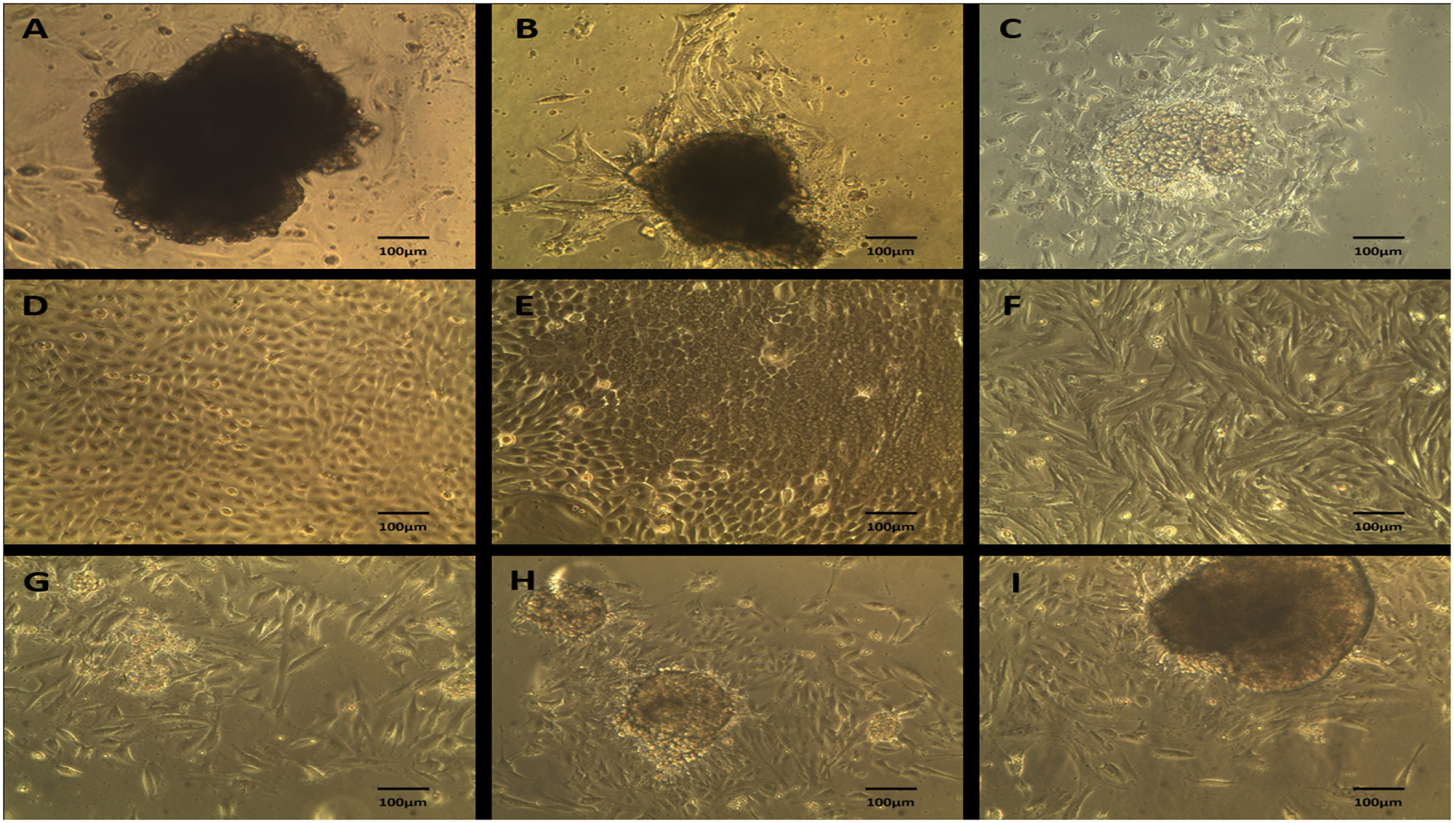
Morphology of primary mixed cell cultures. A-C. Mixed primary culture (organoid) derived from mixed carcinoma, 100x magnification. D-E. Epithelial cells derived by differential trypsinization from adenoma, cultured in Advanced MEM supplemented with mammary epithelial growth supplement (MEGS), 100x magnification. F. Mesenchymal cells (fibroblasts) derived by differential trypsinization from mixed carcinoma, cultured in Advanced MEM supplemented with 10% FBS, 200x magnification. G-I. Organoid derived from normal breast tissue by enzymatic dissociation with collagenase and hyaluronidase IV, 100x magnification.

Primary cells from mammary tumors and normal mammary tissue were isolated by differential trypsinization (Fig 1). Resulting cell cultures were composed by cells with varied morphology, including mixed primary cell population (organoid), epithelial and mesenchymal cells (Fig 2). All experiments were performed on the adherent component of the primary mixed cell cultures after having been submitted three times to differential trypsinization as reported in *Isolation of Primary Mixed Cell Cultures from Spontaneous Mammary Lesions in Canines* section.

**Fig 3:**
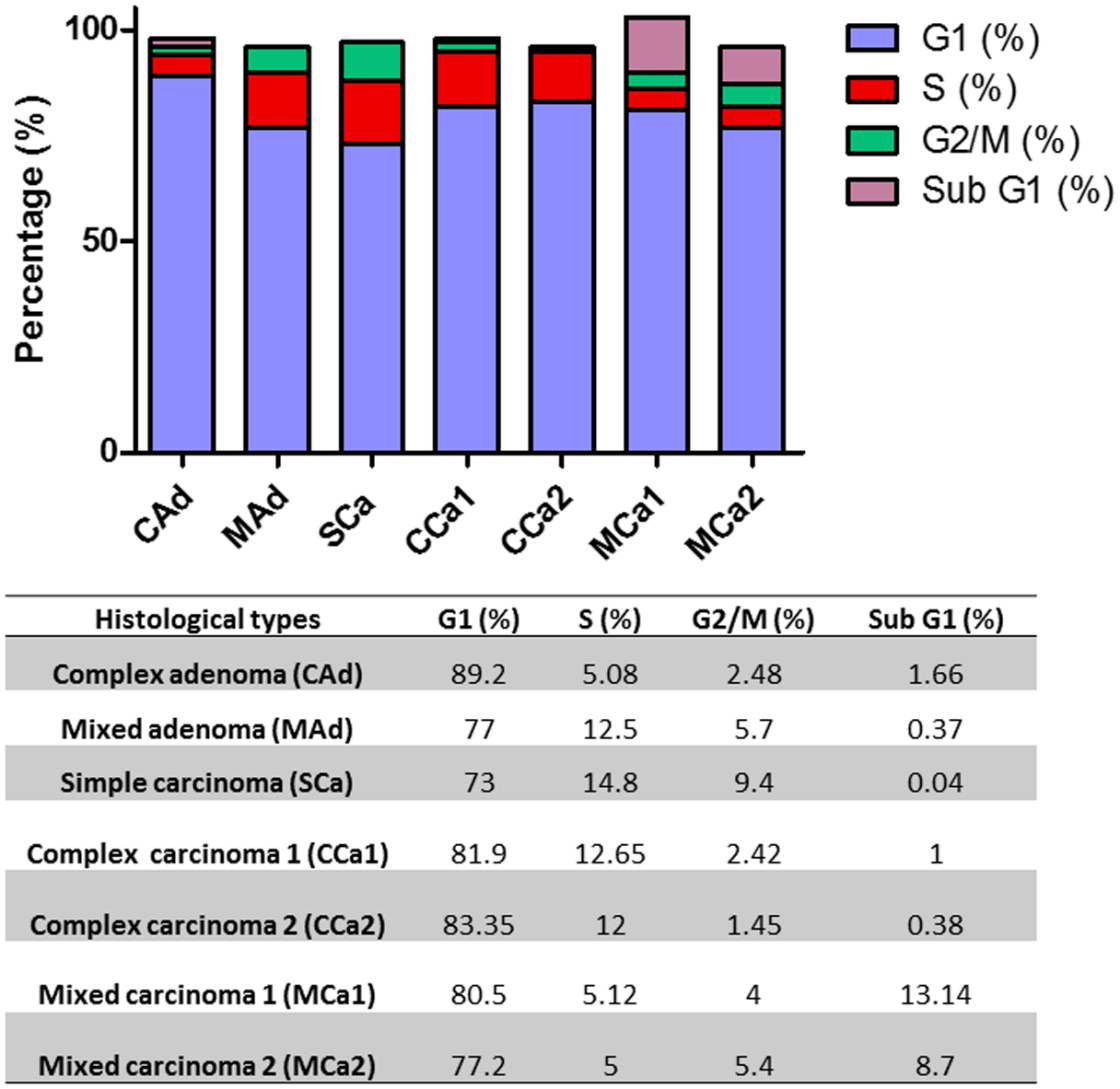
Cell cycle analysis for cells derived from canine mammary gland lesions. Cell cycle phases sub G1, G1, S and G2/M and sub G1 are represented. Table below shows values indicating the percentage of the cell population for each phase of the cell cycle. Complex Adenoma (CAd), Mixed Adenoma (MAd), Simple Carcinoma (SCa), Complex Carcinoma 1 (CCa1), Complex Carcinoma 2 (CCa2), Mixed Carcinoma 1 (MCa1), Mixed Carcinoma 2 (MCa2).

The evaluation of the cell cycle has revealed very similar proliferation profiles for the mixed carcinomas with approximately 80% of cells in G1 phase and 10% of the cells in sub G1 phase; for the complex carcinomas with nearly 80% of cells in G1 and 10% of cells in S phase. In paralel, mixed adenoma and simple carcinoma presented similarity with 75% of the cells in G1 phase and 14% of cells in S phase, whereas complex adenoma had 89% of the cells in G1 phase and 5% of the cells in S phase (Fig 3).

**Fig 4:**
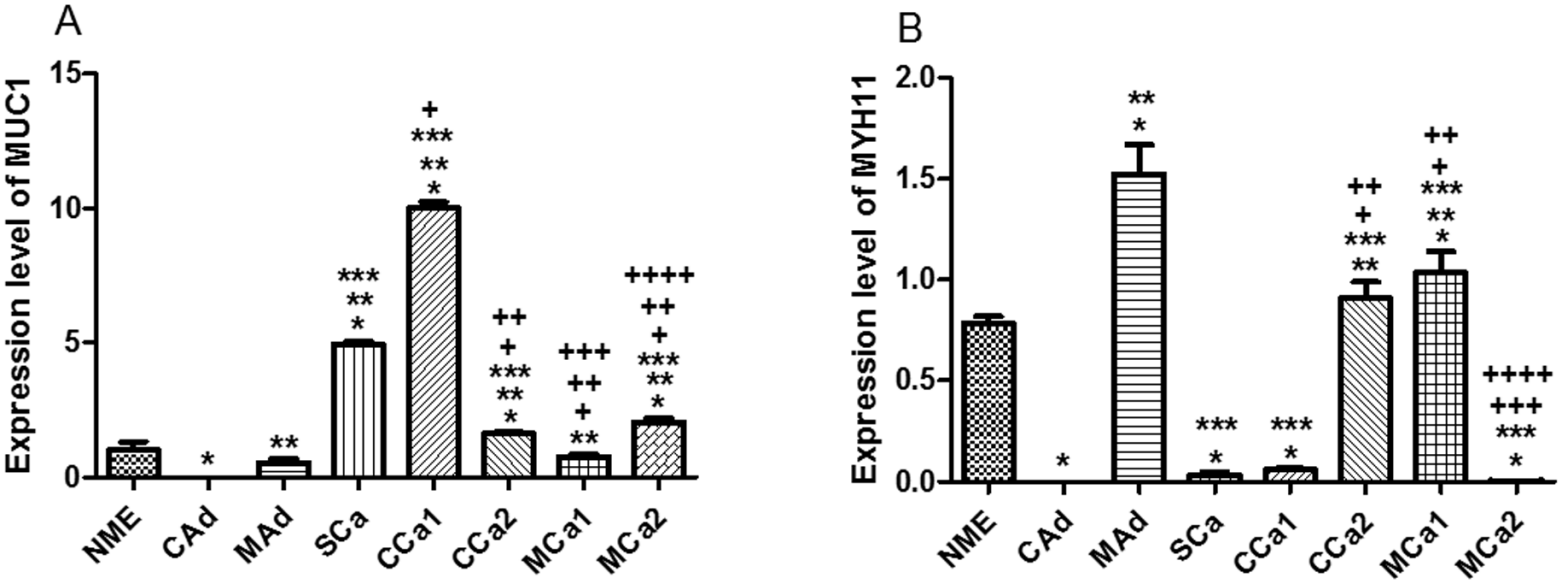
Expression level of mRNA in primary mixed cell cultures obtained from tissue tumor samples. *A*. MYH11 where * *P* < 0.05 versus NME, ** *P* < 0.05 versus CAd, *** *P* < 0.05 versus MAd, + *P* < 0.05 versus SCa, ++ *P* < 0.05 versus CCa1, +++ *P* < 0.05 versus CCa2, ++++ *P* < 0.05 versus MCa1. *B*. MUC1 where * *P* < 0.05 versus NME, ** *P* < 0.05 versus CAd, *** *P* < 0.05 versus MAd, + *P* < 0.05 versus SCa, ++ *P* < 0.05 versus CCa1, +++ *P* < 0.05 versus CCa2, ++++ *P* < 0.05 versus MCa1. Normal Mammary Epithelium (NME), Complex Adenoma (CAd), Mixed Adenoma (MAd), Simple Carcinoma (SCa), Complex Carcinoma 1 (CCa1), Complex Carcinoma 2 (CCa2), Mixed Carcinoma 1 (MCa1), Mixed Carcinoma 2 (MCa2).

Expression levels of a marker for a characteristic epithelial cell surface associated mucin (MUC1) and of a specific smooth muscle marker of myoepithelial cells, the myosin heavy chain 11 (MYH11) were evaluated to analyze the phenotype of primary mixed cell cultures. The epithelial marker, MUC1, had its highest levels in simple and complex carcinoma 1, while the smooth muscle marker, MYH11, has presented its highest expression levels in mixed adenoma, complex carcinoma 2 and mixed carcinoma 1 (Fig 4A and 4B). It is interesting to observe that all cell cultures presented both epithelial and mioepithelial cells with different ratios. In descending order, the expression level of MUC1 transcripts was complex carcinoma 1, simple carcinoma and mixed carcinoma 2. In descending order, the expression level of MYH11 mRNA was mixed adenoma, mixed carcinoma 1, complex carcinoma 2 and normal mammary epithelial (Fig 4A and 4B). Analysis of the ratio between epithelial and myoepithelial markers in all samples indicated an inverse correlation between epithelial and myoepithelial markers in normal mammary epithelium: higher expression of MYH11 transcripts in MAd; higher expression of MUC1 transcripts in SCa; higher expression of MUC1 transcripts in CCa1; balanced amount of epithelial and myoepithelial markers in CCa2 and MCa1, and higher expression of MUC1 transcripts in MCa2. These results show that complex and mixed carcinomas have different ratios within the same tumor subtype, raising the possibility that the prevalence of one cell type on the other could interfere in the behavior of those primary mixed cell cultures.

**Fig 5:**
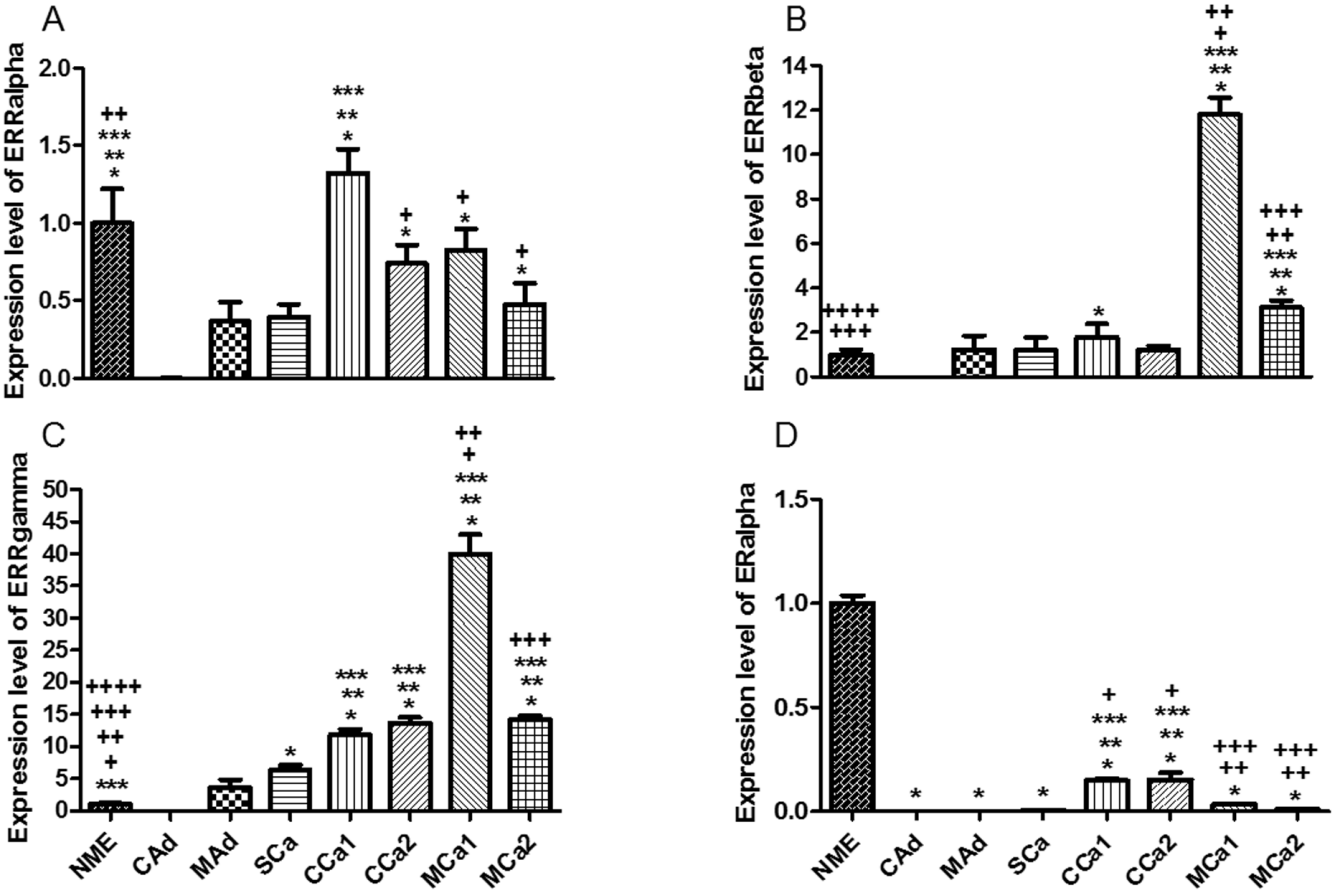
Expression level of mRNA in primary mixed cell cultures obtained from tissue tumor samples. *A*. ERRα where * *P* < 0.05 versus CAd, ** *P* < 0.05 versus MAd, *** *P* < 0.05 versus SCa, + *P* < 0.05 versus CCa1, ++ *P* < 0.05 versus MCa2. *B*. ERRβ where * *P* < 0.05 versus CAd, ** *P* < 0.05 versus MAd, *** *P* < 0.05 versus SCa, + *P* < 0.05 versus CCa1, ++ *P* < 0.05 versus CCa2, +++ *P* < 0.05 versus MCa1, ++++ *P* < 0.05 versus MCa2. *C*. ERRγ where * *P* < 0.05 versus CAd, ** *P* < 0.05 versus MAd, *** *P* < 0.05 versus SCa, + *P* < 0.05 versus CCa1, ++ *P* < 0.05 versus CCa2, +++ *P* < 0.05 versus MCa1, ++++ *P* < 0.05 versus MCa2. *D*. ERα where * *P* < 0.05 versus NME, ** *P* < 0.05 versus CAd, *** *P* < 0.05 versus MAd, + *P* < 0.05 versus SCa, ++ *P* < 0.05 versus CCa1, +++ *P* < 0.05 versus CCa2. Normal Mammary Epithelium (NME), Complex Adenoma (CAd), Mixed Adenoma (MAd), Simple Carcinoma (SCa), Complex Carcinoma 1 (CCa1), Complex Carcinoma 2 (CCa2), Mixed Carcinoma 1 (MCa1), Mixed Carcinoma 2 (MCa2).

The analysis of the gene expression in all cell lines isolated from the seven mammary tumors revealed the highest expression level of nuclear receptor ERRα in the complex carcinoma 1 (CCa1) sample, while in the rest of the cell lines the levels were lower than in mammary epithelium, with the lowest levels in those cells obtained from the adenomas and simple carcinoma (Fig 5A). Regarding the expression levels for ERRβ and γ, the results showed similar profiles among the cell lines. Both nuclear receptors were highly expressed in the same mixed carcinoma (MCa1). However, transcript levels for ERRγ were higher in complex carcinomas when compared to ERRβ. Interestingly, the transcript levels for ERRγ were the highest among ERRs in all analyzed tissues (Fig 5A–5C). Concerning expression levels of ERα, complex carcinomas presented the highest levels among the tumor samples, whereas all tumors revealed expression lower than that found in mammary epithelium sample (Fig 5D).

**Fig 6:**
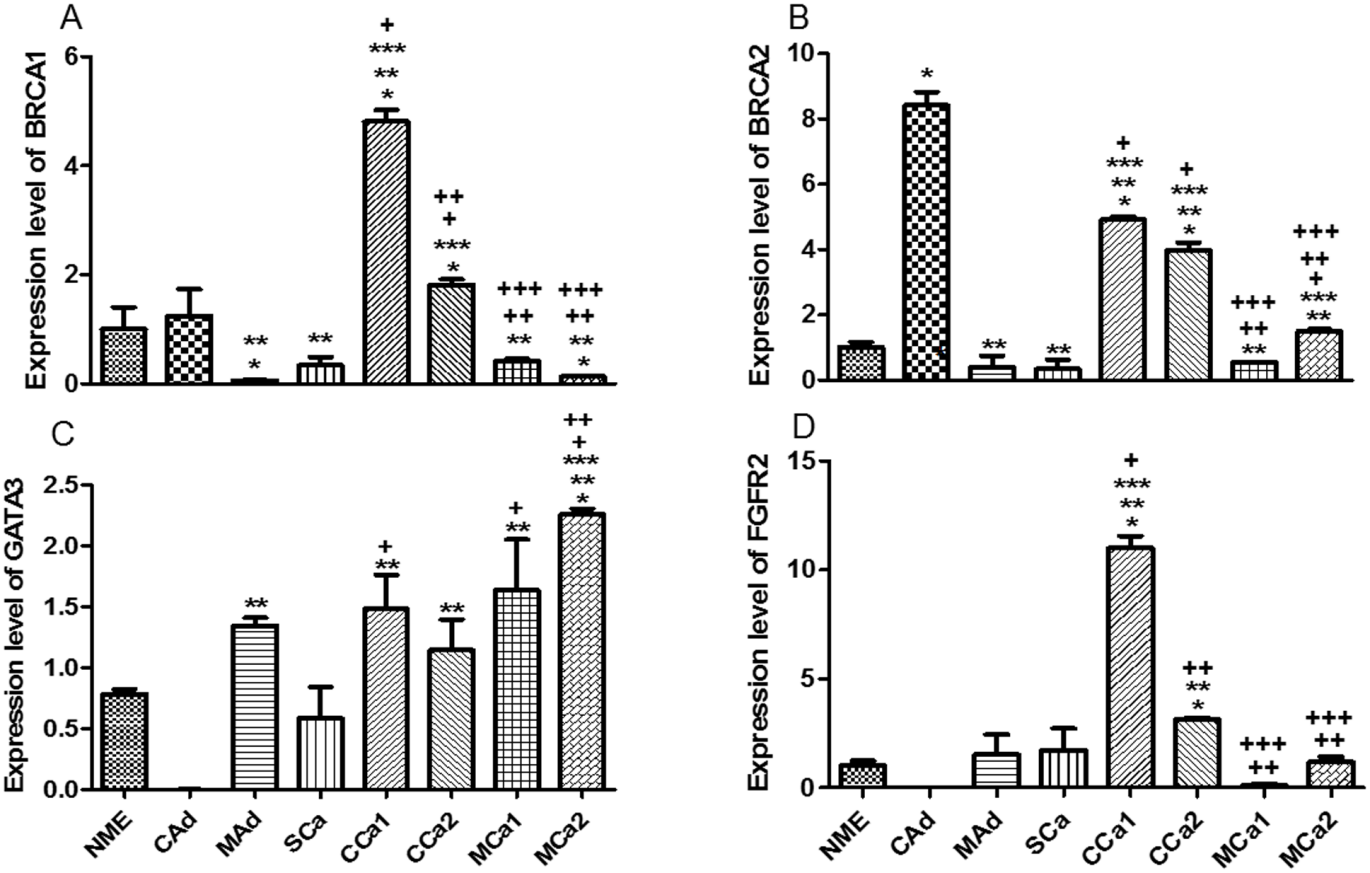
Expression level of mRNA in primary mixed cell cultures obtained from tissue tumor samples. *A*. BRCA1 where * *P* < 0.05 versus NME, ** *P* < 0.05 versus CAd, *** *P* < 0.05 versus MAd, + *P* < 0.05 versus SCa, ++ *P* < 0.05 versus CCa1, +++ *P* < 0.05 versus CCa2. *B*. BRCA2 where * *P* < 0.05 versus NME, ** *P* < 0.05 versus CAd, *** *P* < 0.05 versus MAd, + *P* < 0.05 versus SCa, ++ *P* < 0.05 versus CCa1, +++ *P* < 0.05 versus CCa2. *C*. GATA3 where * *P* < 0.05 versus NME, ** *P* < 0.05 versus CAd, *** *P* < 0.05 versus MAd, + *P* < 0.05 versus SCa, ++ *P* < 0.05 versus CCa2. *D*. FGFR2 where * *P* < 0.05 versus NME, ** *P* < 0.05 versus CAd, *** *P* < 0.05 versus MAd, + *P* < 0.05 versus SCa, ++ *P* < 0.05 versus CCa1. Normal Mammary Epithelium (NME), Complex Adenoma (CAd), Mixed Adenoma (MAd), Simple Carcinoma (SCa), Complex Carcinoma 1 (CCa1), Complex Carcinoma 2 (CCa2), Mixed Carcinoma 1 (MCa1), Mixed Carcinoma 2 (MCa2).

*BRCA1* and *2* highest expression levels were verified in the complex carcinomas samples and in complex adenoma exclusively for *BRCA2* (Fig 6). The transcription factor GATA3 had its highest levels in mixed carcinoma samples and its lowest levels in complex adenoma (Fig 6C). The transcript expression levels for FGFR2 showed the highest values for the complex carcinoma samples (Fig 6D).

**Fig 7:**
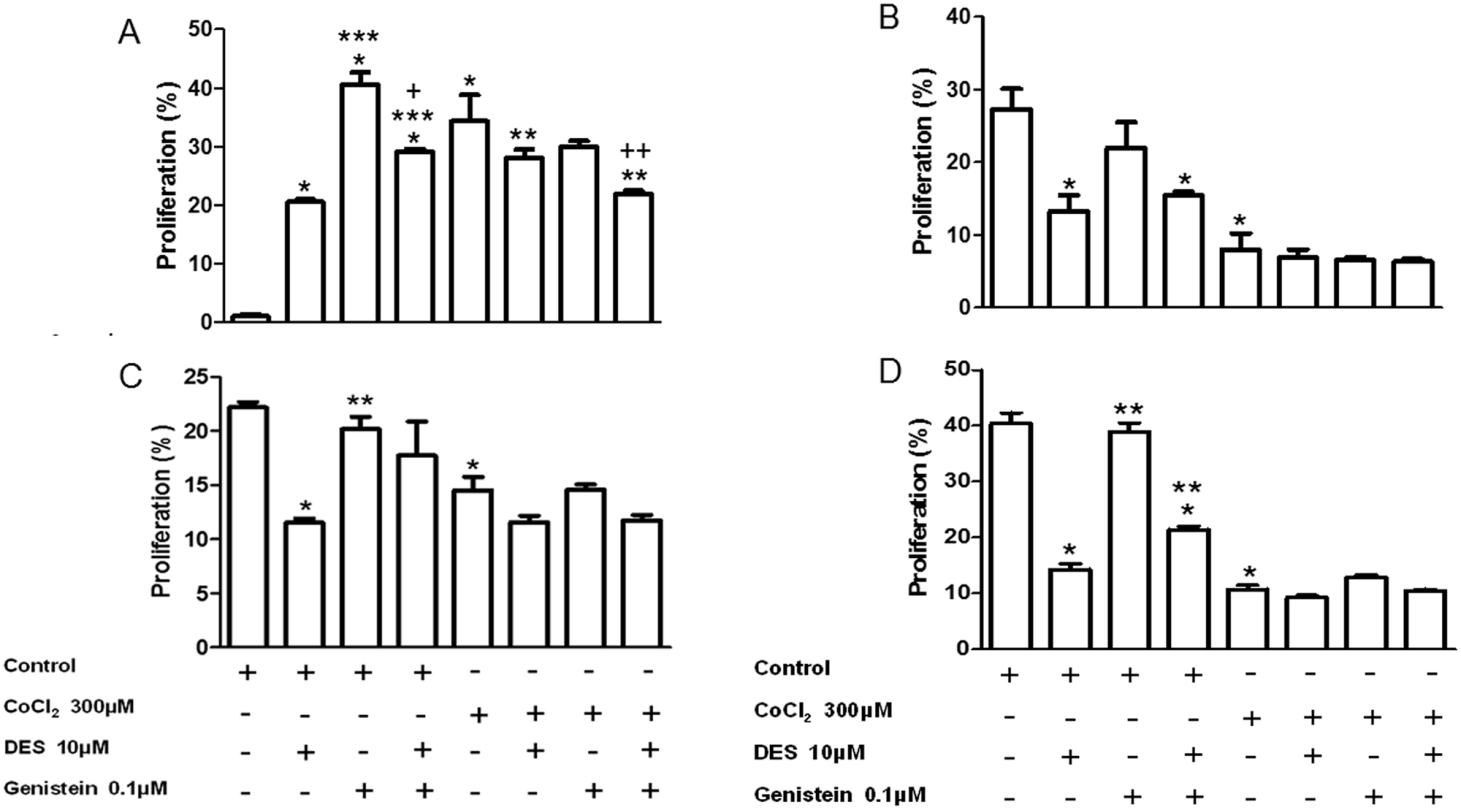
Proliferation assays in primary mixed cell cultures. DES and genistein treatment effect on the basal proliferation and proliferation in the presence of CoCl_2_ cells derived from NME (A), SCa (B), CCa1 (C) and CCa2 (D). Proliferation was assessed 48 h after treatments. The graphics represent an independent experiment with the mean and standard deviation of triplicates. A. * P < 0.05 versus control, ** *P* < 0.05 versus CoCl_2_, *** *P* < 0.05 versus DES 10μM, + *P* < 0.05 versus genistein 0.1μM, ++ *P* < 0.05 versus genistein + CoCl_2_. B. * P < 0.05 versus control. C. * P < 0.05 versus control, ** *P* < 0.05 versus DES 10μM. D. * P < 0.05 versus control, ** *P* < 0.05 versus DES 10μM. Normal Mammary Epithelium (NME), Simple Carcinoma (SCa), Complex Carcinoma 1 (CCa1), Complex Carcinoma 2 (CCa2).

According to the results obtained from the analysis of proliferation of four primary mixed cell cultures, in cells derived from normal mammary tissue, there was an observed increase of cell proliferation with the three treatments (DES, genistein and genistein plus DES) in normoxic/basal conditions. The treatment with genistein induced highest levels of proliferation (40%) and when DES is added to this treatment the proliferative levels were decreased to 30%. In addition, treatment with the chemical hypoxia inducer cobalt chloride (CoCl_2_) increased the proliferation of normal mammary cells while the treatment with DES partially inhibited this proliferation and the treatment with genistein potentialized the inhibition effect of DES (Fig 7A, S3 Fig). Regarding the proliferation of cells derived from the simple carcinoma sample, data showed that cells obtained from simple carcinoma had 30-fold higher proliferation rate than normal cells and, under normoxic condition, the treatment with DES inhibited this proliferation while there was no effect of genistein on this parameter. In addition, in hypoxic conditions, there was a decrease in the proliferation of these cells in absence or presence of pharmacological modulators (Fig 7B, S4 Fig). The two complexes carcinoma samples proved to be somewhat similar in behavior, proliferating more than normal cells. In the cell culture derived from complex carcinoma 1, there was a decrease in basal proliferation with DES treatment. Interestingly, when those cells were treated with DES and genistein the inhibitory effect of DES on proliferation was blocked. Decrease in cell proliferation was observed in hypoxic conditions, but none of the treatments induced change in the proliferative rate in those conditions (Fig 7C, S5 Fig). The behavior of cells derived from complex carcinoma 2 (CCa2) sample was like that described above with the difference that, in addition to treatment with DES, DES and the treatment with genistein also inhibited cell proliferation showing no effect of genistein on the cellular proliferation in presence of DES (Fig 7D, S6 Fig).

## Discussion

In veterinary medicine studies, description of ERRs roles is so far restricted to the development of the mammary gland in cattle where the expression of ERα, PR, ERRα and ERRβ was assessed by qPCR and immunohistochemistry in calves and cows in various stages of lactation and reproduction. Elevated levels of ERRα, ERα and PR were observed in all physiological stages of mammary gland in cattle, in contrast to very low levels of ERRβ expression, suggesting a common regulation of the top three nuclear receptors in the physiology of the mammary gland [42]. Concerning canine mammary gland pathologies, such as neoplasias, there are no scientific records clarifying the role of ERRs in this specie. For this reason, the discussion was conducted comparing the findings in breast cancer human patients, as well as experimental studies, with the present results in female dogs.

Adenoma is considered a benign neoplasm of myoepithelial or well-differentiated epithelial cells, whereas carcinomas are considered malignant neoplasms and are classified into several types, including simple carcinoma, complex carcinoma and mixed carcinoma. Simple carcinomas present only malignant proliferation of epithelial cells while complex carcinomas present, besides malignant proliferation of epithelial cells, presence of benign proliferation of myoepithelial cells. Finally, mixed carcinomas display malignant proliferation of epithelial cells, with benign proliferation of myoepithelial cells and presence of osseous and/or cartilage metaplasia [38, 39].

The gene expression analysis of ERRα in eight cell cultures obtained from one normal mammary tissue, used as a control, and seven mammary tumors revealed the highest expression level of this nuclear receptor in a complex carcinoma sample (CCa1). The lowest levels of ERRα transcripts, meanwhile, were observed in adenomas and simple carcinoma. Regarding the expression levels for ERRβ and γ, the results have shown similar profiles among the cell lines. Both nuclear receptors were highly expressed in the same mixed carcinoma (MCa1). However, the transcript levels of ERRγ were higher in complex carcinomas when compared to ERRβ. Interestingly, the transcript levels for ERRγ were the highest between ERRs in all analyzed tissues.

The importance of ERRs in human breast cancer has been studied by comparing their mRNA profiles with clinically established pathological signs, ER mRNA profiles and members of the ErbB family. The mRNA levels of ERalpha, ERbeta, epidermal growth factor receptor, ErbB2, ErbB3, ErbB4, ERRα, ERRβ, and ERRγ were determined in unselected primary breast tumors and normal mammary epithelial cells enriched from reduction mammoplasties. ERRα showed potential as a biomarker of unfavorable clinical outcome and, possibly, hormonal insensitivity. Increased ERRα levels were associated with ER-negative and PgR-negative tumor status [28, 29]. ERRα levels also correlated with expression of ErbB2, an indicator of aggressive tumor behavior [28]. Unlike ERRα, ERRγ showed potential as a biomarker of favorable clinical course and, possibly, hormonal sensitivity. ERRγ was overexpressed in 75% of the tumors. ERRγ overexpression was associated with hormonally responsive ER- and PgR-positive status. In addition, ERRγ expression correlated with levels of ErbB4, a likely indicator of preferred clinical course, and associated with diploid-typed tumors. Hence, ERRα and ERRγ status may be predictive of sensitivity to hormonal blockade therapy, and ERRα status may also be predictive of ErbB2-based therapy such as herceptin [28]. The role of ERRγ in human invasive breast cancers obtained by radical mastectomy was acessed by immunohistochemical analysis. Nuclear immunoreactivity of ERRγ was detected in 79% of the cases and correlated with the lymph node status. No significant associations were observed with other clinicopathological characteristics, including the expression levels of both estrogen and progesterone receptors [43]. Thus, considering the histopathological classification, the results of this study indicate that higher levels of expression of ERRα are found in samples of carcinomas while the expression level was the lowest in those cells obtained from complex and mixed adenomas, a fact which corroborates the stipulations of the literature indicating this receptor as a marker related to neoplasms of poor prognosis, such as carcinomas.

Regarding the expression levels for ERRβ and γ mRNA, the results have shown a similar profile for the receptors in the primary mixed cell cultures obtained from mixed carcinomas (MCa1 e MCa2). Although the transcript levels for ERRγ were higher when compared to ERRβ, both nuclear receptors display the same expression pattern in the two mixed carcinomas. The role of ERRγ in breast cancer is controversial. One study demonstrated ERRγ-induced resistance to tamoxifen (TAM) in ER+ breast cancer models, and that the transcriptional activity of this receptor is modified by activation of the ERK/MAPK pathway [44]. The same research group also determined whether ERRγ target genes were associated with reduced distant metastasis-free survival (DMFS) in ER+ breast cancer treated with TAM. The data indicated ERRγ signaling was associated with poor DMFS in ER+, TAM-treated breast cancer, suggesting hyper-activation or over-expression of ERRγ induces a pro-survival transcriptional program that impairs the ability of TAM to inhibit the growth of ER+ breast cancer [45]. On the other hand, ERRγ has been implicated in the activation of expression of a genetic program characteristic of mesenchymal to epithelial transition (MET) with induction of E-cadherin and suppression of cancer growth, conferring a protective role for this nuclear receptor against cancer invasiveness [46]. The data obtained in this study appear to indicate no correlation between ERRγ and ERα, with low expression levels for ERα and high expression levels for ERRγ in all studied mixed cell cultures.

Overexpression of ERRβ in breast cancer patients is correlated with improved prognosis and longer relapse-free survival, and the level of ERRβ mRNA is inversely correlated with the S-phase fraction of cells from breast cancer patients. ERRβ also acts as a potential tumor-suppressor molecule in breast and prostate cancer [47, 48]. The cascade of ERRβ signalling events may lead to blockage of the G1/S transition and inhibition of the epithelial to mesenchymal transition (EMT) through regulation of E-cadherin [47]. Regarding the inverse correlation between S-phase and ERRβ expression level, the results confirm the data in literature with mixed cell cultures obtained from mixed carcinomas presenting the highest expression levels of the nuclear receptor and the lowest S-phase fraction.

Considering the set of scientific evidence regarding ER and ERRs, the present data demonstrates that, at least in the analyzed samples, all mixed cell cultures presented expression levels of ERα lower than expression levels of ERRα and ERRγ, showing no clear correlation between either higher expression of ERRα in tumors with low levels of ER transcripts or higher expression of ERRγ and high expression of ER. Indeed, data have shown ERα and ERRα display quite the same expression pattern in mixed cell cultures obtained from complex carcinomas (CCa1 and CCa2) with the highest expression levels of both receptors among the tumors. Also, in all malignant tumors, the ERR that is most expressed is ERRγ, indicating no apparent dualism between the ERRs isoforms as it has been seen in humans.

Carcinomas that exhibit reduced expression of BRCA1 are typically poorly differentiated with high proliferative rate, and inversely correlated with histologic grade [49]. In dogs, the study of the expression of the BRCA1 gene demonstrated that loss of BRCA1 expression was associated with increased Ki67 proliferation marker and the absence of estrogen receptor (ER) and other malignancy [50]. Although hereditary mutations in BRCA1 gene are associated with familial breast cancer, the majority of the mammary carcinomas present sporadic occurrence. Although mutations in the BRCA1 are rare in non-familial mammary carcinomas, it is common to have loss of expression in 40 to 50% of sporadic mammary tumors, possibly through epigenetic alterations, specifically promoter hypermethylation [49]. In this study, the results of qPCR indicated that the highest levels of BRCA1 are presented in a complex carcinoma (CCa1), while cultured cells obtained from a complex adenoma showed the highest levels of BRCA2, followed by complex carcinomas. All those mixed cell cultures presented low values for G2/M-phase: 2.5 for CAd, 2.4 for CCa1, and 1.45 for CCa2. Also, the lowest expression levels for BRCA1 were verified in MAd, SCa, MCa1 and MCa2, which are mixed cell cultures obtained from low expression ER and high G2/M-phase percentage tumors, corroborating data found in the literature.

Experimental studies in mice have shown that loss of GATA-3 expression is associated with high histologic grade tumors [37]. So far, there are no expression or epigenetic studies about GATA-3 transcription factor in dogs. The results indicate that lower levels of GATA3 were observed in complex adenoma and simple carcinoma, while the highest levels of expression were between the complex and mixed carcinomas, tumors with high histologic grade.

The tyrosine kinase receptors comprise a family of signaling molecules involved in various physiological processes regulating cell proliferation, differentiation and apoptosis, which are commonly dysregulated in cancer [51, 52]. Alterations in FGFR pathways such as amplifications and overexpression are noted in 5 to 30% of mammary tumors in women. In a preclinical study conducted on human breast carcinomas from 58 patients, it was observed that the action of 17-β-estradiol altered the expression and/or localization of FGFR2 and that 62% of the tumor cells showed moderate to intense FGFR2 labeling in their cytoplasm [51]. Several authors have reported the occurrence of single nucleotide polymorphisms (SNPs) in FGFR2 as a risk factor for breast cancer in women and the study of their expression with prognostic value in relation to survival and disease-free period, and its increased expression is associated with poor clinical outcome [52, 53, 54]. There are, to date, no studies of FGFR2 expression in mammary neoplasms in the canine species. The current results showed mixed cell cultures obtained from complex carcinomas presented the highest expression levels of FGFR2, while the complex adenoma sample presented the lowest expression level. Therefore, canine FGFR2 appears to correlate with the same receptor in humans in terms of increased expression in unfavorable prognosis tumors. However, further studies analyzing SNPs would be helpful to provide additional information about the biological role of this receptor in canine mammary cancer.

Genistein is among the main soy isoflavones and presents structural and functional similarities to 17-β-estradiol and can bind estrogen receptors alpha (ERα) and beta (ERβ). This explains its relationship to the phytoestrogen family, a class of non-steroidal phytochemicals which act like estrogen-like compounds [55]. Due to the structural resemblance with 17-β-estradiol, genistein mediates most of its biological effects through the modulation of ER signaling pathways. In hormone-dependent tissues, estrogens play an important role in many physiological processes, such as cell proliferation, differentiation or apoptosis. However, high levels of estrogens are a major risk factor for the development of hormone-dependent diseases, such as breast or prostate cancer. It is still not completely clear why endogenous or synthetic estrogens increase breast cancer risk, while phytoestrogens, structurally similar compounds, appear to have the opposite effect [56]. Genistein has been shown to inhibit cell proliferation in both ER-positive and ER-negative breast cancer cell lines [57]. It induces apoptosis and G2/M cell cycle arrest and decreases cell motility [58]. Genistein has also been shown to play a role in several signaling pathways indicated in carcinogenesis, reducing the expression and/or activation of several procarcinogenic proteins, such as cyclin D1, Akt, and erbB2 and increasing the expression of the tumor suppressor, phosphatase and tensin homologue (PTEN) [56].

Diethylstilbestrol (DES) is a synthetic nonsteroidal estrogen which was prescribed for women from 1947 until 1971 to prevent spontaneous abortions. However, not only was the drug therapeutically ineffective in reducing the incidence of miscarriages, but reports have established that women exposed in utero to DES showed an increased incidence of a rare type of vaginal cancer [59, 60]. A recent study showed that in utero DES-exposed women greater than 40 years of age exhibited a statistically significant increase in the relative risk of developing breast cancer [61].

Considering these antagonic features of DES and genistein, four mixed cell cultures were selected for functional proliferation assay: the culture obtained from normal mammary tissue (NME) and, due to the malignancy and absence of metaplasia, the culture derived from the simple carcinoma (SCa) and two cultures obtained from compex carcinomas (CCa). The effect of DES and/or genistein on basal proliferation and hypoxia was observed in mixed cultures derived from normal mammary tissue, simple carcinoma and complex carcinoma. Various treatments were effective in reducing the proliferation of cells grown from simple and complex carcinomas. Considering the inhibitory action of genistein and the stimulatory effect of DES on neoplasic proliferation data have shown the evaluated mixed cell cultures did respond to those drugs in a different manner. Probably the differences in those responses are due to the action of these pharmaceuticals on ERRs and further studies are necessary to disclose the molecular mechanisms evolved in this process.

Regarding the role of ERRα in proliferative capacity of the cell lines derived from simple carcinoma and complex carcinomas, the blocking of the three isoforms of ERRs, with the DES treatment [28], leads to decreased basal proliferation of all three cell lines. However, treatment with the ERRα agonist, genistein [28], was not effective in modifying the proliferation rate of these cells. Still, genistein treatment has not shown any synergistic effect with DES treatment. These results indicate that, at least directly, ERRα does not stimulate cell proliferation in these cell lines, and that this stimulatory function of proliferation may be performed by another isoform of the receptor (ERRγ or β).

Taken together, the results point to a differentiated role of ERRγ in canine mammary cancer, where its expression appears to be increased in mixed carcinomas, followed by complex carcinomas. Concerning the proliferation of neoplastic cells, ERRα seems to not be implicated in the stimulation of the proliferation rate in these cells.

## Supporting information

S1 Fig Supporting information: Cell cycle analysis. Cells were first gated in dot plot cytograms of size (FSC—forward scatter) and internal complexity (SSC—side scatter), excluding debris. Doublet discrimination was then performed in dot plots cytograms of PI fluorescence in FL2-A and FL2-W. Finally, using FL2-H linear histograms, FlowJo cell cycle tool was requested to calculate the cell percentage in each cycle phase. We used the Watson pragmatic algorithm.
(TIF)

S2 Fig Supporting information: Ploidy analysis. Cells were firstly gated in dot plot cytograms of size (FSC—forward scatter) and internal complexity (SSC—side scatter), excluding debris. Then, doublet discrimination was performed in dot plots cytograms of PI fluorescence in FL2-A and FL2-W. Finally, using FL2-H linear histograms, cell lines geometric mean of propidium iodide (PI) staining fluorescence was calculated and divided by lymphocytes’ geometric mean of fluorescence, thus generating a ploidy index.
(TIF)

S3 Fig Supporting information: Representative images of NME cells proliferation analysis by flow cytometry. Dot plot cytogram depicts gating of viable and intact cells, excluding debris, by size (FSC—forward scatter) and internal complexity (SSC—side scatter). Histograms for green fluorescence (FL1 channel) demonstrate CFSE staining dilution due to proliferation in 48 h. Each histogram is representative of one replicate value for each treatment condition, performed in triplicate.
(TIF)

S4 Fig Supporting information: Representative images of SCa cells proliferation analysis by flow cytometry. Dot plot cytogram depicts gating of viable and intact cells, excluding debris, by size (FSC—forward scatter) and internal complexity (SSC—side scatter). Histograms for green fluorescence (FL1 channel) demonstrate CFSE staining dilution due to proliferation in 48 h. Each histogram is representative of one replicate value for each treatment condition, performed in triplicate.
(TIF)

S5 Fig Supporting information: Representative images of CCa1 cells proliferation analysis by flow cytometry. Dot plot cytogram depicts gating of viable and intact cells, excluding debris, by size (FSC—forward scatter) and internal complexity (SSC—side scatter). Histograms for green fluorescence (FL1 channel) demonstrate CFSE staining dilution due to proliferation in 48 h. Each histogram is representative of one replicate value for each treatment condition, performed in triplicate.
(TIF)

S6 Fig Supporting information: Representative images of CCa2 cells proliferation analysis by flow cytometry. Dot plot cytogram depicts gating of viable and intact cells, excluding debris, by size (FSC—forward scatter) and internal complexity (SSC—side scatter). Histograms for green fluorescence (FL1 channel) demonstrate CFSE staining dilution due to proliferation in 48 h. Each histogram is representative of one replicate value for each treatment condition, performed in triplicate.
(TIF)
