# Supplementary figures and images for: Establishment of primary mixed cell cultures from spontaneous canine mammary tumors: Characterization of classic and new cancer-associated molecules
Source: PLoS One. 2017 Sep 25;12(9):e0184228. doi: 10.1371/journal.pone.0184228 (PMC5612463; doi:10.1371/journal.pone.0184228)

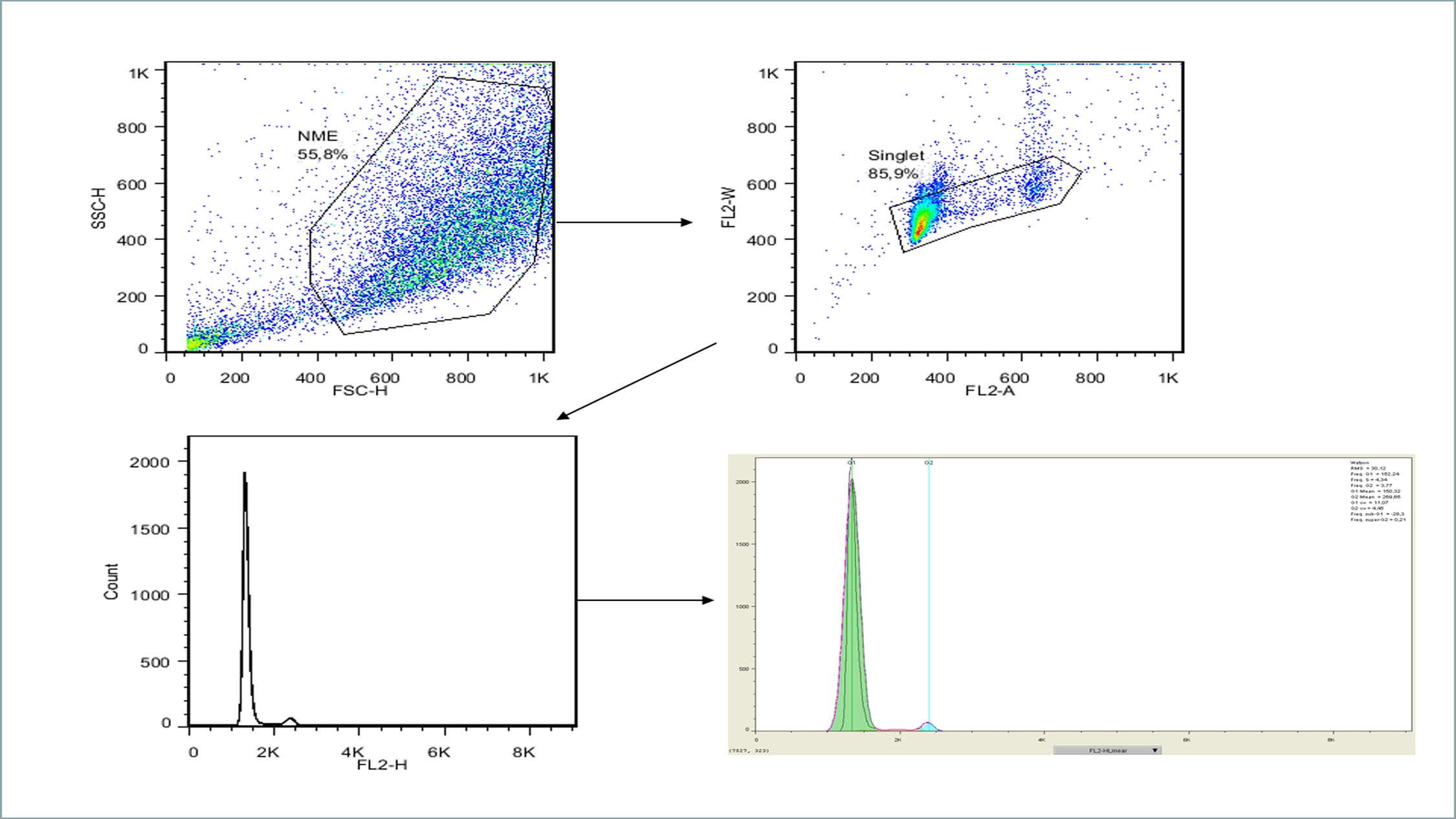

Supplement: S1 Fig — Cells were first gated in dot plot cytograms of size (FSC—forward scatter) and internal complexity (SSC—side scatter), excluding debris. Doublet discrimination was then performed in dot plots cytograms of PI fluorescence in FL2-A and FL2-W. Finally, using FL2-H linear histograms, FlowJo cell cycle tool was requested to calculate the cell percentage in each cycle phase. We used the Watson pragmatic algorithm. (TIF) [file pone.0184228.s001.tif]

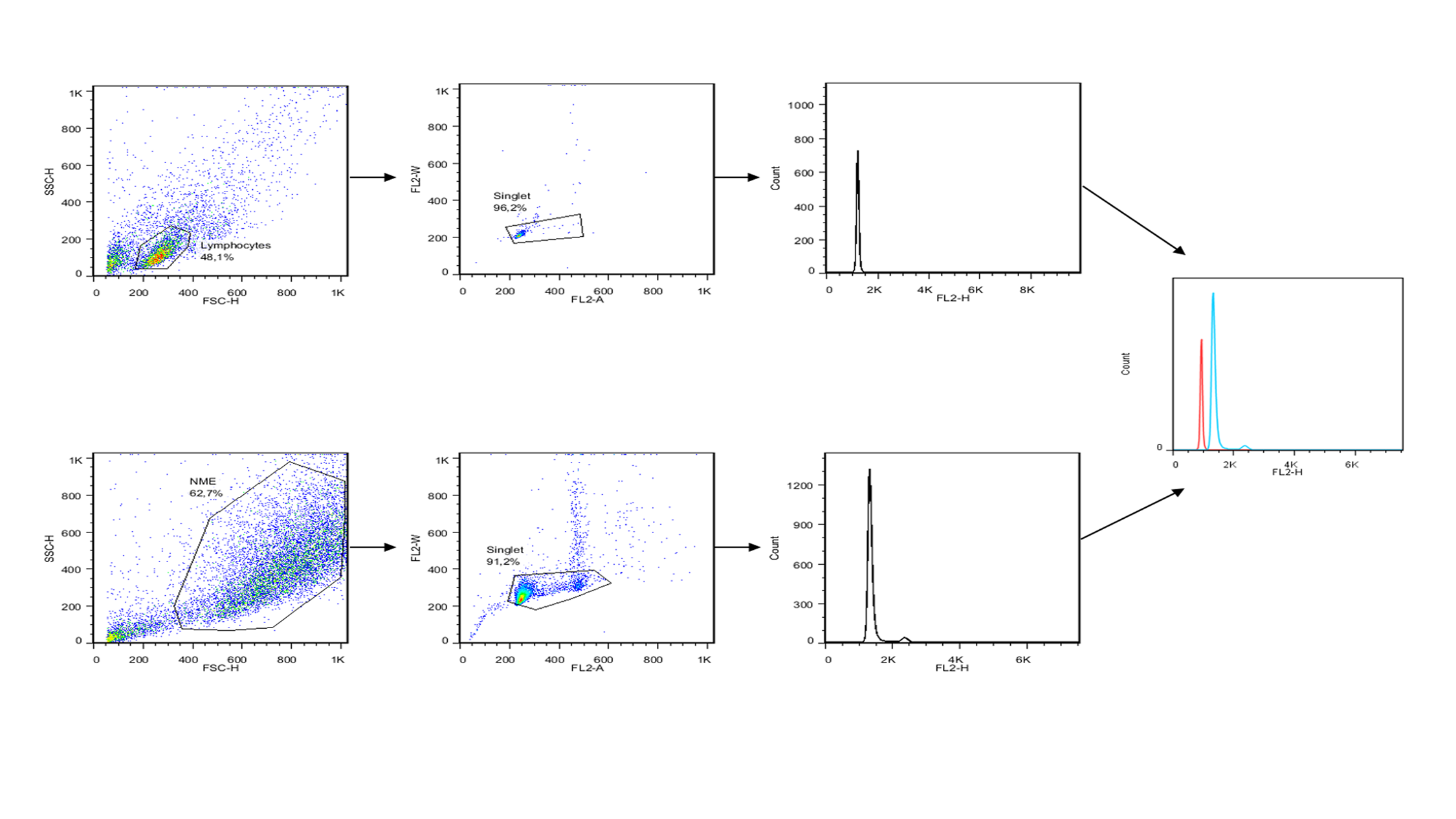

Supplement: S2 Fig — Cells were firstly gated in dot plot cytograms of size (FSC—forward scatter) and internal complexity (SSC—side scatter), excluding debris. Then, doublet discrimination was performed in dot plots cytograms of PI fluorescence in FL2-A and FL2-W. Finally, using FL2-H linear histograms, cell lines geometric mean of propidium iodide (PI) staining fluorescence was calculated and divided by lymphocytes’ geometric mean of fluorescence, thus generating a ploidy index. (TIF) [file pone.0184228.s002.tif]

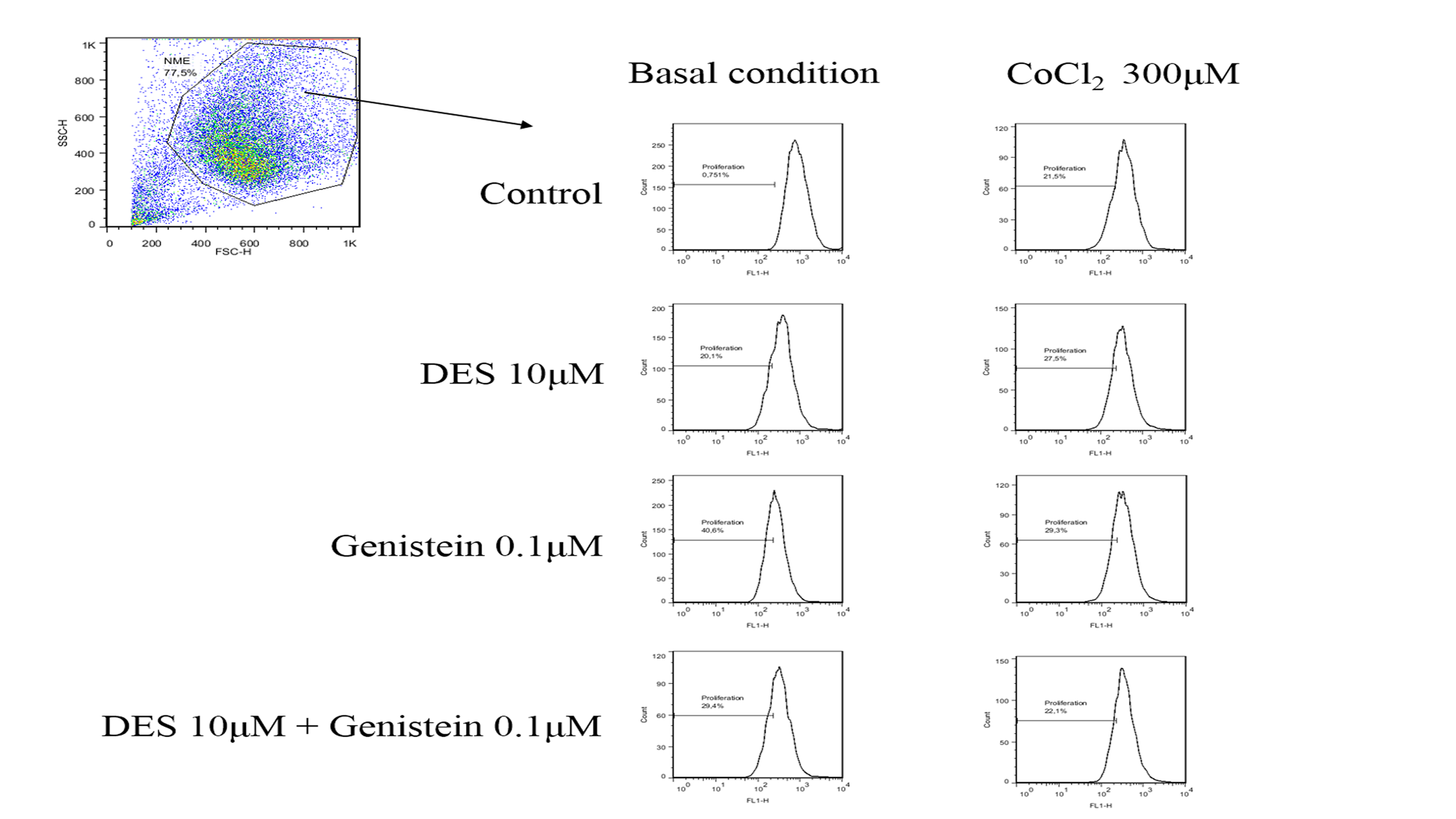

Supplement: S3 Fig — Dot plot cytogram depicts gating of viable and intact cells, excluding debris, by size (FSC—forward scatter) and internal complexity (SSC—side scatter). Histograms for green fluorescence (FL1 channel) demonstrate CFSE staining dilution due to proliferation in 48 h. Each histogram is representative of one replicate value for each treatment condition, performed in triplicate. (TIF) [file pone.0184228.s003.tif]

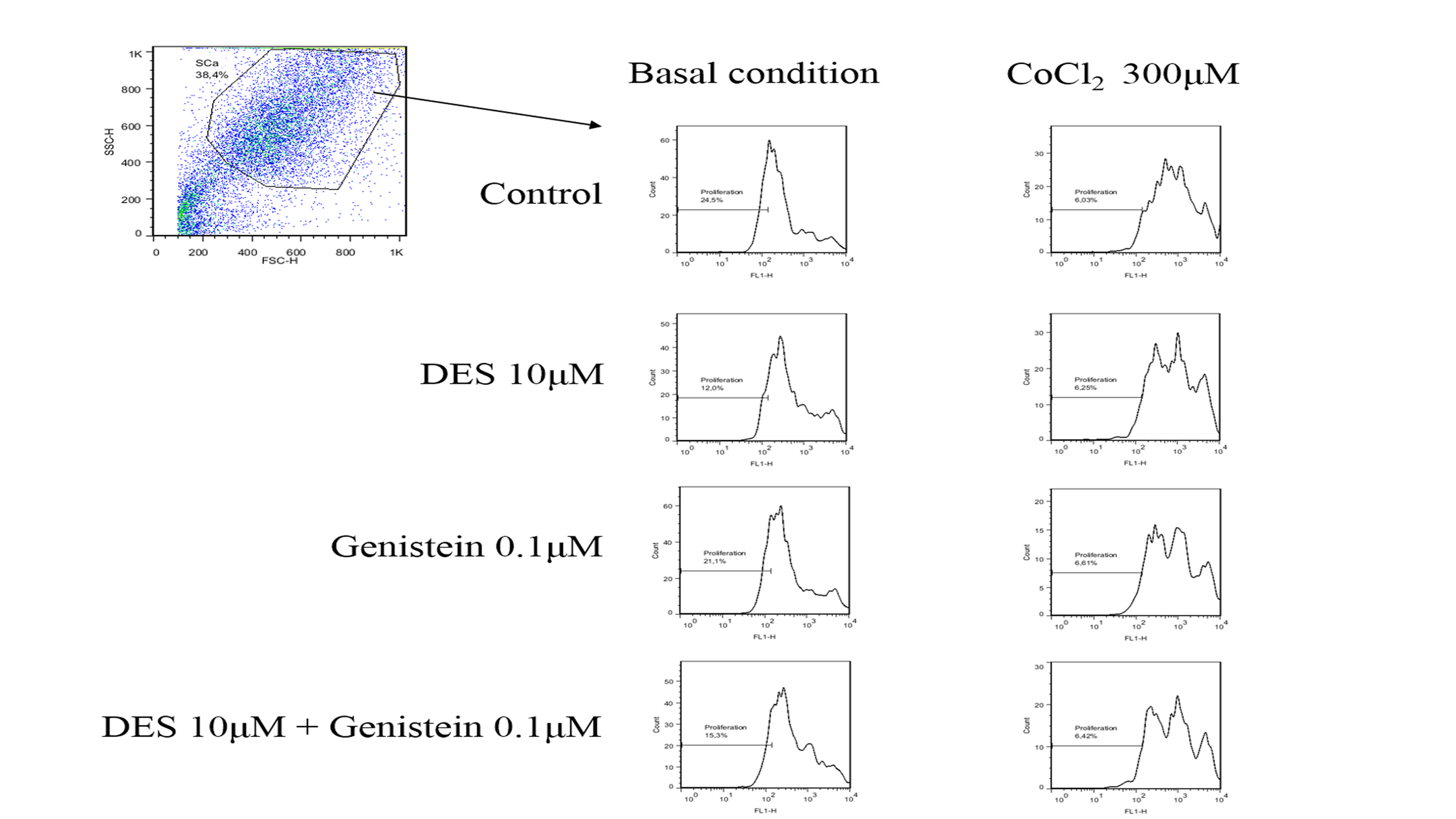

Supplement: S4 Fig — Dot plot cytogram depicts gating of viable and intact cells, excluding debris, by size (FSC—forward scatter) and internal complexity (SSC—side scatter). Histograms for green fluorescence (FL1 channel) demonstrate CFSE staining dilution due to proliferation in 48 h. Each histogram is representative of one replicate value for each treatment condition, performed in triplicate. (TIF) [file pone.0184228.s004.tif]

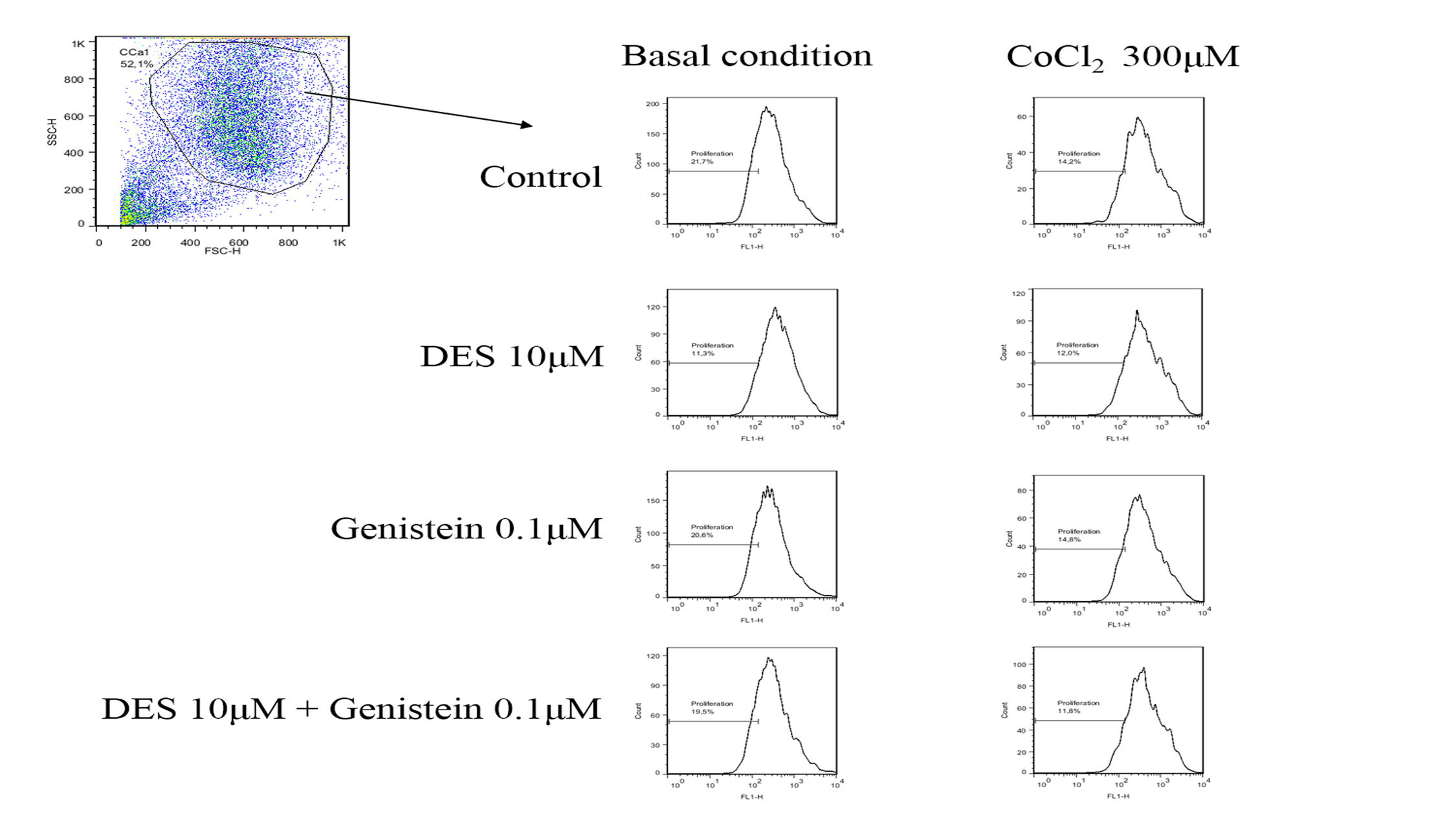

Supplement: S5 Fig — Dot plot cytogram depicts gating of viable and intact cells, excluding debris, by size (FSC—forward scatter) and internal complexity (SSC—side scatter). Histograms for green fluorescence (FL1 channel) demonstrate CFSE staining dilution due to proliferation in 48 h. Each histogram is representative of one replicate value for each treatment condition, performed in triplicate. (TIF) [file pone.0184228.s005.tif]

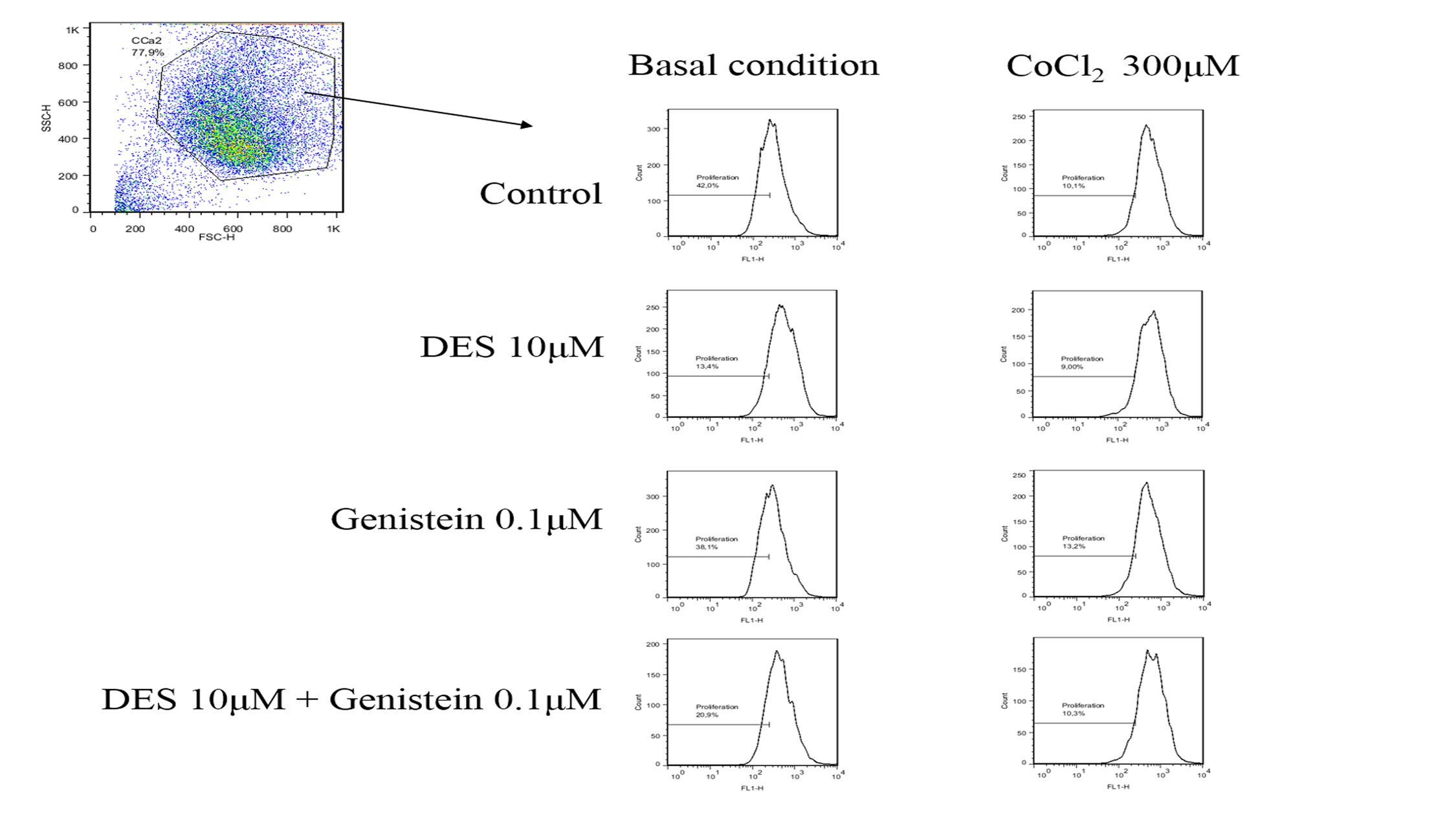

Supplement: S6 Fig — Dot plot cytogram depicts gating of viable and intact cells, excluding debris, by size (FSC—forward scatter) and internal complexity (SSC—side scatter). Histograms for green fluorescence (FL1 channel) demonstrate CFSE staining dilution due to proliferation in 48 h. Each histogram is representative of one replicate value for each treatment condition, performed in triplicate. (TIF) [file pone.0184228.s006.tif]
